# Measuring public transport accessibility to fixed activities and discretionary opportunities: a space–time approach

**DOI:** 10.1186/s12544-024-00636-2

**Published:** 2024-01-31

**Authors:** Alberto Dianin, Michael Gidam, Georg Hauger, Elisa Ravazzoli

**Affiliations:** 1 Faculty of Architecture and Spatial Planning, Vienna University of Technology, Karlsgasse 11, 1040 Vienna, Austria; 2 Eurac Research, Institute for Regional Development, Viale Druso 1, 39100 Bolzano, Italy

**Keywords:** Accessibility, Public transport, Space–time model, Fixed activities, Discretionary opportunities

## Abstract

The Space–Time Accessibility (STA) model is broadly used to measure person-based accessibility based on the space, time, and transport constraints experienced at the individual level in connection to the actual modal choices of observed individuals. In this paper, we propose to adjust the STA model (by introducing a so-called PT-STA model) with three core purposes: (1) focusing on public transport accessibility specifically, (2) measuring accessibility to both fixed activities and discretionary opportunities, and (3) integrating travel-time thresholds in the accessibility measurement. These three elements aim to make the PT-STA model a suitable tool to evaluate the impacts of public transport interventions on person-based accessibility and extend the evaluation of public transport accessibility to its fixed and discretionary dimensions. The PT-STA model is tested with a sample of 118 residents of the rural municipality of Mühlwald (South Tyrol, Italy). Results show that the accessibility to fixed activities and discretionary opportunities are limitedly correlated. It is possible to identify people with high accessibility to fixed activities and poor access to discretionary opportunities (typically pensioners and homemakers living in remote locations) and vice versa (e.g. people with articulated rural–urban commutes but daily visiting locations with several amenities). These results preliminarily confirm the importance of combining both accessibility dimensions in the PT-STA model since they tell complementary and not overlapped stories that are relevant for policymakers to evaluate the pros and cons of alternative public transport interventions.

## Introduction

Accessibility measures, in general, can be grouped into “place-based” and “person-based” approaches [30]. While the former focus on the accessibility of a place by assuming that all people experience the same accessibility, the latter investigates how accessibility varies across people in the same area depending on their individual space, time, and transport constraints [19, 25]. As such, person-based measures are broadly used to investigate accessibility differences across (groups of) individuals, which is a key topic for the transport equity debate [15] and for practitioners who increasingly aim to make transport systems not only effective and efficient but even fair [43, 44]. Space–time accessibility models (from now on STA), are among the most diffused person-based accessibility measures [16, 29]. They can measure accessibility individually based on the space–time constraints that each person experiences due to e.g. their employment, household composition, age, gender, or degree of access to different transport means. Many studies have used STA models to detect accessibility variations in society (such as [24, 27, 36]). Nevertheless, the traditional STA measure also has some downsides (e.g. [11, 26, 31]). In this study, we intend to focus on three of them.

First, the STA typically focuses on the transport mode(s) people use daily to perform their fixed activities [16]. From the perspective of policymakers, it would be useful to deploy the STA even to measure person-based accessibility by specific modes that can be objects of planning interventions, such as transit, biking, or carpooling [8]. This is particularly important considering that EU-level targets, such as the Sustainable Development Goals, also call for public transport improvements that should support all groups of society and improve the fairness of the transport system [42]. Second, the STA typically focuses on how many discretionary opportunities people may access to, given the spatiotemporal constraints placed by their daily schedules [25]. Conversely, they only say a little about how easily people can access their mandatory activities [11]. Considering that many EU policies aim to ease access to e.g. work and education using collective and active modes [12], integrating both the fixed and discretionary dimensions in the STA is relevant to get a more complete picture of person-based accessibility. Third, the STA focuses on what is doable for people in space and time based only on their schedules. As such, people with few constraints may tend to register high accessibility even if they have poor transport systems at their disposal (e.g. [15]). Travel-time thresholds may be integrated into the STA to deal with this issue and exclude from the analysis opportunities that, although reachable based on the schedules, cannot be reached within reference thresholds. This is particularly relevant for space–time models since they deal with services and opportunities that all society members should reach within a reasonable effort to achieve sufficient well-being and quality of life [12].

Based on these limits, this study aims to adjust the traditional STA model with three purposes: (1) focusing on public transport (PT) accessibility, (2) measuring accessibility to both fixed activities and discretionary opportunities; and (3) integrating travel-time thresholds in the accessibility measurement. To focus on accessibility by PT only, the model adjusts the way of defining the space–time path of fixed activities and introduces a so-called “PT Reachability Index” to weigh the results. To measure the access to fixed activities and discretionary opportunities, the model produces two key output indicators reflecting these two accessibility dimensions. Both output indicators integrate travel-time thresholds by considering reference standards set at EU and national levels (see Sect. 2 for more details). The model is tested with a sample of 118 individuals living in the rural municipality of Mühlwald (South Tyrol, Italy). By developing the model, this study aims to integrate the traditional space–time approach to enable a deeper PT accessibility evaluation, which can serve as a basis to assess person-based accessibility differences at the status quo, as well as implications of PT interventions on such differences.

The article is structured as follows. Section 2 presents the adaptation of the STA model for this paper. Section 3 tests the model with the sample defined above. Section 4 discusses the results to identify the complementary and added value of the proposed model, as well as its limits. Section 5 concludes the paper by focusing on the potential usage of our model in the evaluation of PT interventions.

## Model

### The standard form of space–time accessibility

**Fig. 1:**
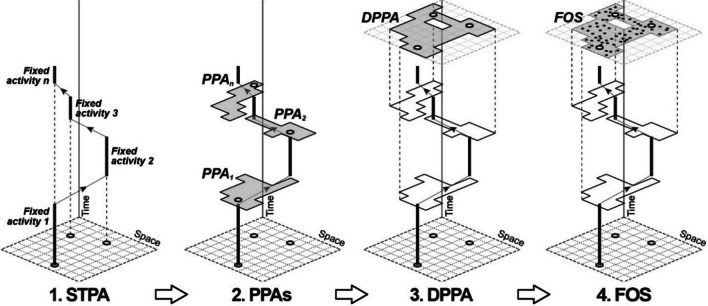
The four steps of the standard STA model

The STA stems from the time geography framework [18], and it is one of the most diffused measures of person-based accessibility, together with the utility-based measure [16, 25], given its high sensitiveness to transport, space and time constraints experienced at the individual level [31]). Operationally, the STA measures the set of additional discretionary opportunities (e.g. retail, pharmacies and other facilities) that individuals could visit on a typical weekday given the constraints posed by their fixed activity chain (e.g. work, mandatory errands or education) and the transport system at their disposal. This set is called Feasible Opportunity Set (FOS) and is reached in four steps (Fig. 1). First, the daily sequence of mandatory and discretionary activities typically performed by a person in a standard weekday is reconstructed (usually through a travel-diary survey). The mandatory activities constrained in space and time (called “fixed”) are isolated from the discretionary ones, forming a scheme of the daily duties. This scheme is called Space–Time Path (STPA). For each couple of subsequent fixed activities in the STPA, the locations an individual could visit given the ending time of the former fixed activity, the starting time of the last fixed activity, the necessary travel time between them, and the time required to visit such opportunities are identified. These locations form a Potential Path Area (PPA) for each fixed activity pair. All the PPAs are merged to obtain the Daily Potential Path Area (DPPA). Finally, all the discretionary opportunities within the DPPA are counted, forming the FOS (i.e., the key output of STA).

### The PT-STA model

To focus on the PT accessibility to fixed activities and discretionary opportunities and integrate the travel-time thresholds, the classic STA formulation needs to be adjusted. The PT-STA model presented in the following subsection proposes such an adjustment in five modelling steps. Three of them (1–3) deal with the setup of the STPA, identification of the PPAs, and composition of the FOS. The remaining two (4–5) regard the calculation of the two output indicators of the model, i.e. the Space–Time Accessibility to fixed activities (STA^PT^_fix_) and discretionary opportunities (STA^PT^_dis_), both integrating travel-time thresholds.

**Fig. 2:**
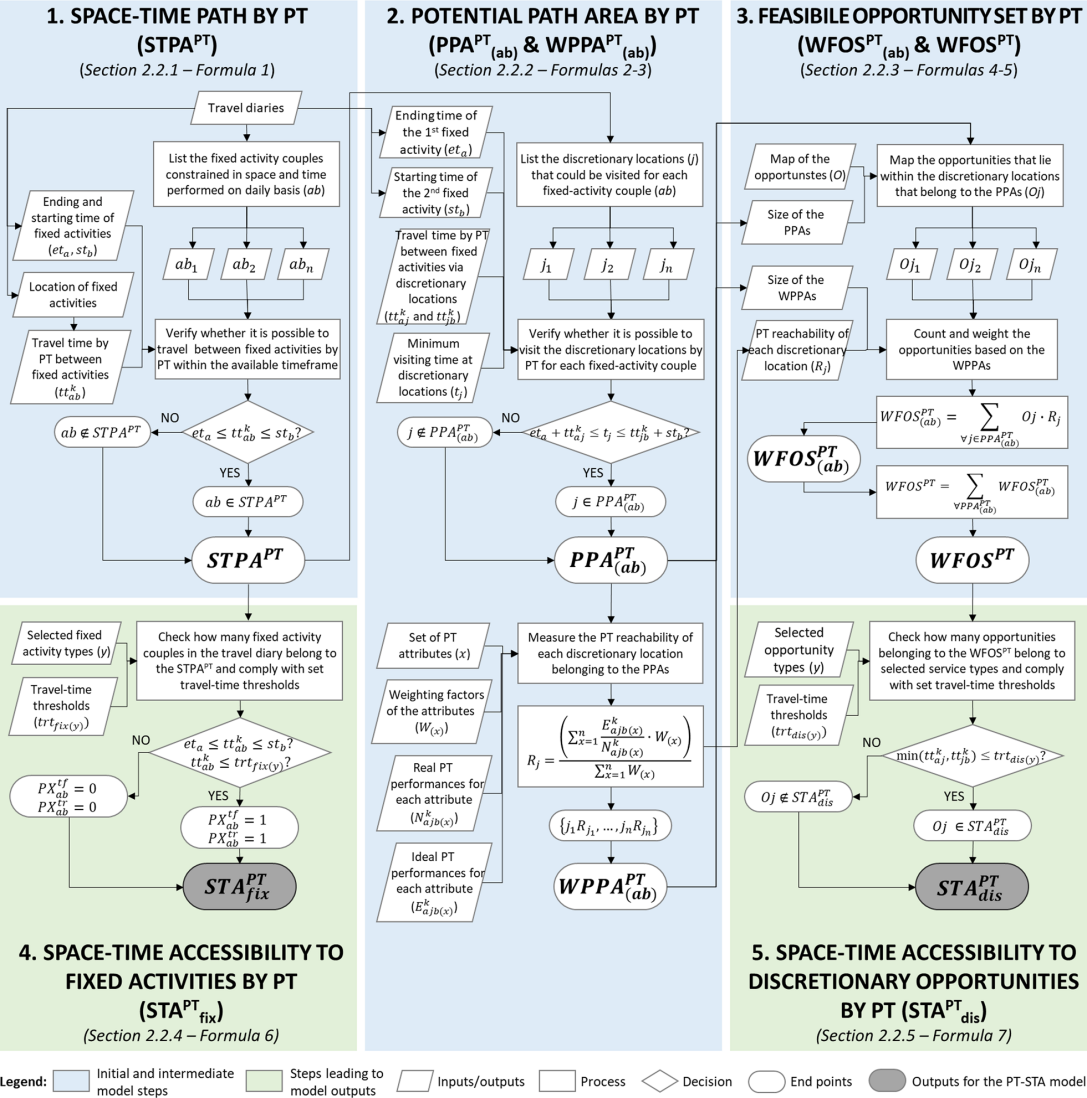
Flowchart of the PT-STA model

**Fig. 3:**
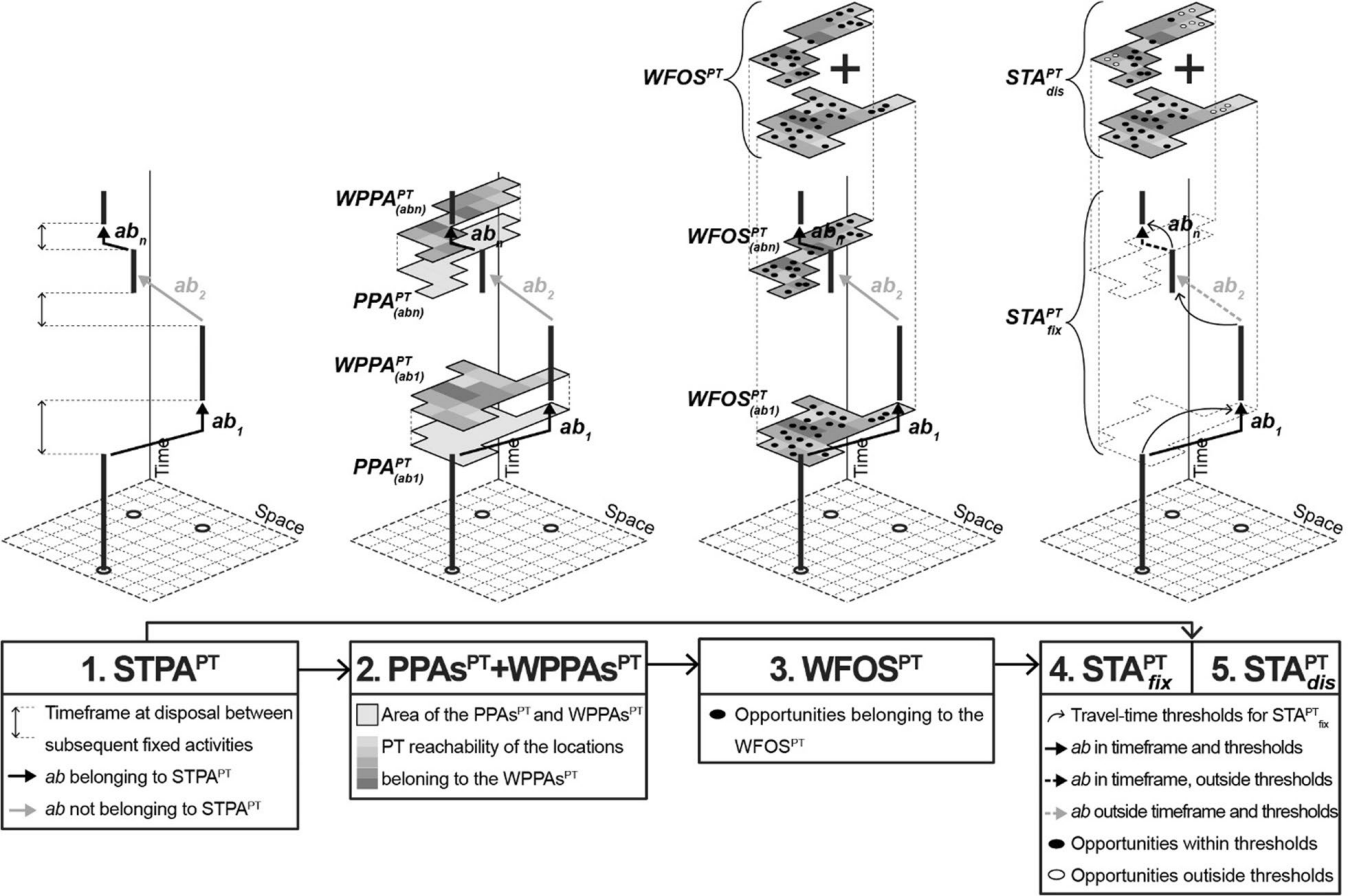
The five steps of the PT-STA model

Figure 2 provides a flowchart of the five calculation steps of the PT-STA model (1–5) including their data sources. These steps are presented in detail in the Sects. 2.2.1–5. Moreover, at the end of Sect. 2, Fig. 3 schematizes the operational logic of the PT-STA model and allows comparing it to the standard STA logic (presented in Fig. 1).

#### Space–time path by public transport

The STPA^PT^ (Formula 1) grounds on all the fixed activity couples performed by individuals on a typical weekday. The data is obtained from individuals thanks to completing a travel diary questionnaire [41]. Respondents list all the fixed and discretionary activities they perform on a typical weekday. They specify the location where they take place, their starting time, ending time, duration, and activity category (from a predefined set of options).^1^ The timing has to be defined based on the requirements posed by the activity itself, and not based the usual mode of transport. To distinguish fixed and discretionary activities, respondents are asked to label each activity as either “fixed” or “discretionary” based on the following input definition given by the interviewer: “a fixed activity is an activity that you have to perform on your typical weekday, usually at the same place and time, with no or very little flexibility”. Therefore, any activities may be labelled as fixed regardless of the indicated category.^2^ This respondent-based evaluation is especially useful when addressing e.g. homemakers or pensioners, who typically have heterogeneous activities that they consider and treat as fixed, although they do not fall into the most typical mandatory categories such as work or education.

Since the focus lies on PT accessibility, the model checks whether the observed individual can travel between each couple of subsequent fixed activities in the given timeframe by PT, considering their location and starting/ending timing. Only the activity couples that PT can connect according to these criteria belong to the STPA^PT^, while the others do not (as *ab*_*2*_ in Fig. 3). This logic implies that modal interdependencies between activity couples are not incorporated in the formation of the *STPA*^*PT*^ and each activity couple is individually evaluated. We adopt this approach to reflect the idea that accessibility measures reveal the potential of a system for interactions [20]; in this case, the potential offered by the PT system in each activity couple (see Sect. 4.2 for a deeper discussion).

1 $$STPA^{PT} = \left\{ {\left( {ab_{1} , \ldots ,ab_{n} } \right){|}et_{a} \le tt_{ab}^{k} \le st_{b} } \right\}$$

where:

*ab*_*1-n*_ are the couples of subsequent fixed activities *a* and *b* daily registered in the travel diaries.

*et*_*a*_ and *st*_*b*_ are the ending time of *a* and the starting time of *b*.

*tt*^*k*^_*ab*_ is the door-to-door travel time from *a* to *b* by mode of transport *k* (in this case PT) in the given timeframe.

#### Potential path area by public transport

For each activity couple belonging to the STPA^PT^, the PPA^PT^ is defined in two steps. The first step is to identify all the discretionary locations that could be visited in the time buffer at disposal (Formula 2). This depends on the ending and starting time of the fixed activities, on the door-to-door travel time by PT, and on the minimum visiting time to enjoy the opportunities available at the discretionary locations. The travel time by PT comprises the first and last link to the PT stops, the waiting time at the stops, the in-vehicle time, and the time spent in transfers. These are estimated considering the variability of the PT supply during the day, and the PT performances provided by the system in the specific timeframe between subsequent fixed activities.

The second step is the weighting of the PPA^PT^ (WPPA^PT^) through a Reachability Index ranging from 0 to 1 (Formula 3), which stems from the relative-network efficiency concept [17]. For each discretionary location belonging to a PPA^PT^, the real performances of the PT system for the connection from *a* (origin) to* j* (discretionary location) and from *j* to* b* (destination) are compared against the ideal performances for the same connection. The PT performances are usually represented by travel time only. In this case, we take into account three PT attributes that influence reachability and can be computed in GIS based on a GTFS^3^ dataset: *total travel time*, only *out-of-vehicle travel time*, and the *travel-chain sections*. We do not include other attributes e.g. info-mobility, tariffs, and ticketing since they do not involve the space–time dimension, which is the key focus of this study. Table 1 displays the attributes with their description and indicators.

**Table 1:** PT attributes for the calculation of the Reachability index

Code	Attribute	Description	Indicator
*x*_*1*_	Total travel time	Total door-to-door travel time (*tt*) needed to travel from *a* to *j* and from *j* to *b* in the available time window	${tt}_{aj}+{tt}_{jb}$
*x*_*2*_	Out-of-vehicle travel time	Only out-of-vehicle travel time (*ott*) needed to travel from *a* to *j* and from *j* to *b* in the available time window. It includes walking, waiting and transfer time	${ott}_{aj}+{ott}_{jb}$
*x*_*3*_	Travel-chain sections	Number of travel-chain sections (*l*) separated by transfers needed to travel from *a* to *j* and from *j* to *b* in the available time window	${l}_{aj}+{l}_{jb}$

Weighting factors are integrated in the Reachability index to measure the relative importance of each PT attribute against the others. In the PT-STA model, the attributes are represented by a proxy ranging from 0.00 (lowest relative importance) to 1.00 (highest relative importance). Weighting factors may be calculated in different ways depending on data availability. Usually, they are obtained either by involving local experts through a Delphi method, or by collecting end users´ preferences through a dedicated survey, or from a mode choice model [37]. In our application of the PT-STA model to the case study of Mühlwald (Sect. 3), we rely on the results of a large survey conducted in South Tyrol with a sample of ca 1800 residents and deriving overall importance rates for several PT attributes [34], see Sect. 3.2 for further details about the estimation of the weighting factors in our case study). The WPPA^PT^ includes all the discretionary locations of the PPA^PT^, weighted by their Reachability Indexes.

2 $$PPA_{{\left( {ab} \right)}}^{PT} = \left\{ {\left( {j_{1} , \ldots ,j_{n} } \right){|}et_{a} + tt_{aj}^{k} \le t_{j} \le tt_{jb}^{k} + st_{b} } \right\}$$

3 $$WPPA_{{\left( {ab} \right)}}^{PT} = \left\{ {\left( {j_{1} R_{{j_{1} }} , \ldots ,j_{n} R_{{j_{n} }} } \right){|}R_{j} = \left( {\mathop \sum \limits_{x = 1}^{n} \frac{{E_{ajb\left( x \right)}^{k} }}{{N_{ajb\left( x \right)}^{k} }} \cdot W_{\left( x \right)} } \right)/\mathop \sum \limits_{x = 1}^{n} W_{\left( x \right)} } \right\}$$

where:

*a* and* b* are the first and second fixed activities of a fixed activity couple.

*j*_*1-n*_ are all the discretionary locations that can be vested between *a* and *b*.

*et*_*a*_ and *st*_*b*_ are the ending time of *a* and the starting time of *b*.

*tt*^*k*^_*aj*_ and *tt*^*k*^_*jb*_ are the door-to-door travel time by mode *k* (i.e. PT) from *a* to *j* and from *j* to *b*.

*t*_*j*_ is the minimum visiting time to enjoy the opportunities available in *j*.

*R*_*j*_ is the Reachability Index of the discretionary location *j*.

*x*_*1-n*_ are the attributes describing the performances of the PT system.

*E*^*k*^_*ajb(x)*_ is the ideal performance of the connection *ajb* by mode *k* (i.e. PT) for the attribute *x*.

*N*^*k*^_*ajb(x)*_ is the real performance of the connection *ajb* by mode *k* (i.e. PT) for the attribute *x*.

*W*_*(x)*_ is the weighting factor for the PT attribute *x* represented by a proxy ranging from 0.00 to 1.00.

#### Feasible opportunity set by public transport

For each PPA^PT^, the opportunities that lie within them are counted and weighted. This leads to a so-called weighted FOS^PT^ for each fixed activity couple (WFOS^PT^_(ab)_; Formula 4). This includes all the discretionary opportunities (like services, shops or leisure facilities) that lie within the locations including the related PPA^PT^. Each opportunity is weighted according to the Reachability index assigned to the location where it lies. The WFOS^PT^ calculated for each fixed activity couple are summed into the overall WFOS^PT^ (Formula 5). As such, this double counts all the opportunities that can be visited more times per day (i.e. that belong to more PPA^PT^) since this allows incorporating the added values given by the possibility of visiting the same opportunity multiple times a day.

4 $$WFOS_{{\left( {ab} \right)}}^{PT} = \mathop \sum \limits_{{\forall j \in PPA_{{\left( {ab} \right)}}^{PT} }} OjR_{j}$$

5 $$WFOS^{PT} = \mathop \sum \limits_{{\forall PPA_{{\left( {ab} \right)}}^{PT} }} WFOS_{{\left( {ab} \right)}}^{PT}$$

where:

*Oj* are the discretionary opportunities that lie within the discretionary locations *j* belonging to the PPA.

*R*_*j*_ are the Reachability indexes of each location *j* belonging to the PPA.

#### Space–time accessibility to fixed activities by public transport

The Space–Time Accessibility to fixed activities by PT (STA^PT^_fix;_ Formula 6) checks how many fixed activity couples daily performed by an individual can be made by PT within the available timeframes and given travel-time thresholds. Such thresholds represent minimum performances that the PT system should guarantee for the access to mandatory activities like work and education.

As such, STA^PT^_fix_ is calculated as an index that ranges from 0 to 1, with 1 indicating that all the fixed activity couples listed in the travel diary can be performed by PT both within the available timeframe, as well as within given travel-time thresholds. The value of STA^PT^_fix_ depends on two proxy values calculated for each fixed activity couple. The first proxy (equal to either 0 or 1) indicates whether a fixed activity couple can be performed by PT within the available timeframe. The second proxy (equal to either 0 or 1) indicates whether a fixed activity couple can be performed by PT within the given threshold. Such threshold varies depending on the type of fixed activity couple under examination. In this model, we consider three types, namely “home-to-work” (and vice versa), “home-to-school” (and vice versa), and “other errands” travels.

Threshold concepts for fixed activities are used in various EU countries to e.g. identify disservices, find weak areas or set refund policies [4, 7]. For instance, the Austrian Pendlerpauschale (commuter allowance) refunds commuters with home-work trips by PT exceeding defined travel-time thresholds. Methodologically, it is hard to establish a priori thresholds fitting any kinds of context. For instance, a threshold of 15 min for home-to-school trips might be reasonable for urban areas with high service concentration and dense PT network. However, it would not be appropriate in a rural county. Therefore, thresholds have to be estimated on a case-by-case basis by following a common approach, i.e. by considering (1) the transport and land-use characteristics of the study area, (2) existing local PT performance standards if available, and (3) the PT goals set by local policy makers. According to this approach, Sect. 3.2 presents the thresholds estimated for our study area of Mühlwald, which is a rural and remote valley.

6 $$STA_{fix}^{PT} = \mathop \sum \limits_{ab = 1}^{n} \left( {PX_{ab}^{tf} + PX_{ab}^{tr} } \right)/2n\;\quad with\;\left\{ {\begin{array}{*{20}l} {PX_{ab}^{tf} = 1 \Leftrightarrow et_{a} \le tt_{ab}^{k} \le st_{b} } \hfill \\ {PX_{ab}^{tf} = 0 \;otherwise} \hfill \\ {PX_{ab}^{tr} = 1 \Leftrightarrow tt_{ab}^{k} \le trt_{fix\left( y \right)} } \hfill \\ {PX_{ab}^{tr} = 0\; otherwise } \hfill \\ \end{array} } \right.$$

where:

*ab*_*1-n*_ are the couples of subsequent fixed activities *a* and *b* daily registered in the travel diaries.

*PX*^*tf*^_*ab*_ is the proxy defining whether the fixed activity couple *ab* can be performed by PT within the available timeframe (*tf*).

*PX*^*tr*^_*ab*_ is the proxy defining whether the fixed activity couple *ab* can be performed by PT within the set travel-time threshold (*tr*).

*et*_*a*_ and *st*_*b*_ are the ending time of *a* and the starting time of *b*.

*tt*^*k*^_*ab*_ is the door-to-door travel time from *a* to *b* by mode of transport *k* (in this case PT) in the given timeframe.

*trt*_*fix(y)*_ is the travel-time threshold for fixed activity couples of type *y*: home-to-work (*y*_*1*_), home-to-school (*y*_*2*_), other errands (*y*_*3*_).

#### Space–time accessibility to discretionary opportunities by public transport

The Space–Time Accessibility to discretionary activities by PT (STA^PT^_dis;_ Formula 7) indicates a pool of discretionary opportunities individuals can reach and visit based on their space–time constraints and within reference travel-time thresholds. These thresholds represent the minimum performances that a PT system should guarantee to all citizens for the access to basic services like groceries and healthcare.

Following this concept, the STA^PT^_dis_ consists of a subset of the WFOS^PT^ that includes only the discretionary opportunities in the WFOS^PT^ that belong to given types of basic services and that can be reached by PT from the origin or from which the destination can be reached within set travel-time thresholds. As in the previous case, these thresholds vary depending on the type of discretionary opportunity under examination. In this model, we focus on three main types of discretionary opportunities and related thresholds, i.e. “basic retail and pharmacies”, “healthcare and general services”, and “leisure and other facilities”.

Like for STA^PT^_fix_, also the thresholds for the access to discretionary opportunities need to be estimated on a case-by-case basis depending on the study area object of analysis. Thus, it is not possible to establish already in the methodology common thresholds fitting any study areas. Nevertheless, EU countries such as the UK have developed such thresholds to deal with minimum basic service access in remote areas [10, 38], as well as EU projects like the ESPON 2020 project PROFECY [14] have used similar kind of standards to identify inner peripheries in the EU [35]. The examples given by these EU-level initiatives may be combined with local directives, policy goals and desk research to reach context-sound thresholds. According to this approach, Sect. 3.2 presents the threshold regarding discretionary opportunities estimated for our study area of Mühlwald.

7 $$STA_{dis}^{PT} = \left\{ {\left( {Oj_{\left( y \right)} R_{j} \in WFOS^{PT} } \right){\text{|min}}\left( {tt_{aj}^{k} ,tt_{jb}^{k} } \right) \le trt_{dis\left( y \right)} } \right\}$$

where:

*Oj*_*(y)*_*R*_*j*_ are the opportunities *Oj* weighted by the Reachability index *R*_*j*_ included in WFOS^PT^ and belonging to the types *y*.

*tt*^*k*^_*aj*_ are *tt*^*k*^_*jb*_ are the travel time from *a* to *j* and from *j* to *b* by mode of transport *k* (i.e. PT) in the given timeframe.

*trt*_*dis(y)*_ is the travel-time threshold for discretionary opportunities of type *y*: basic retail and pharmacies (*y*_*1*_), healthcare and general facilities (*y*_*2*_), and leisure and other facilities (*y*_*3*_).

As anticipated, Fig. 3 summarises the five steps to undertake for the application of the PT-STA model, which can be compared with those of the standard STA (Fig. 1). In particular, it presents a realistic condition where not all the fixed activity couples listed in the travel diary belong to the STPA^PT^ according to the needed travel time by PT against the available timeframe (see *ab*_*2*_). All the other activity couples in the STPA^PT^ produce their own PPA^PT^, WPPA^PT^ and related WFOS^PT^, so showing the total potential accessibility by PT based on the framework space–time constraints.

## Testing the model in Mühlwald (South Tyrol, Italy)

### Study area and sample

**Fig. 4:**
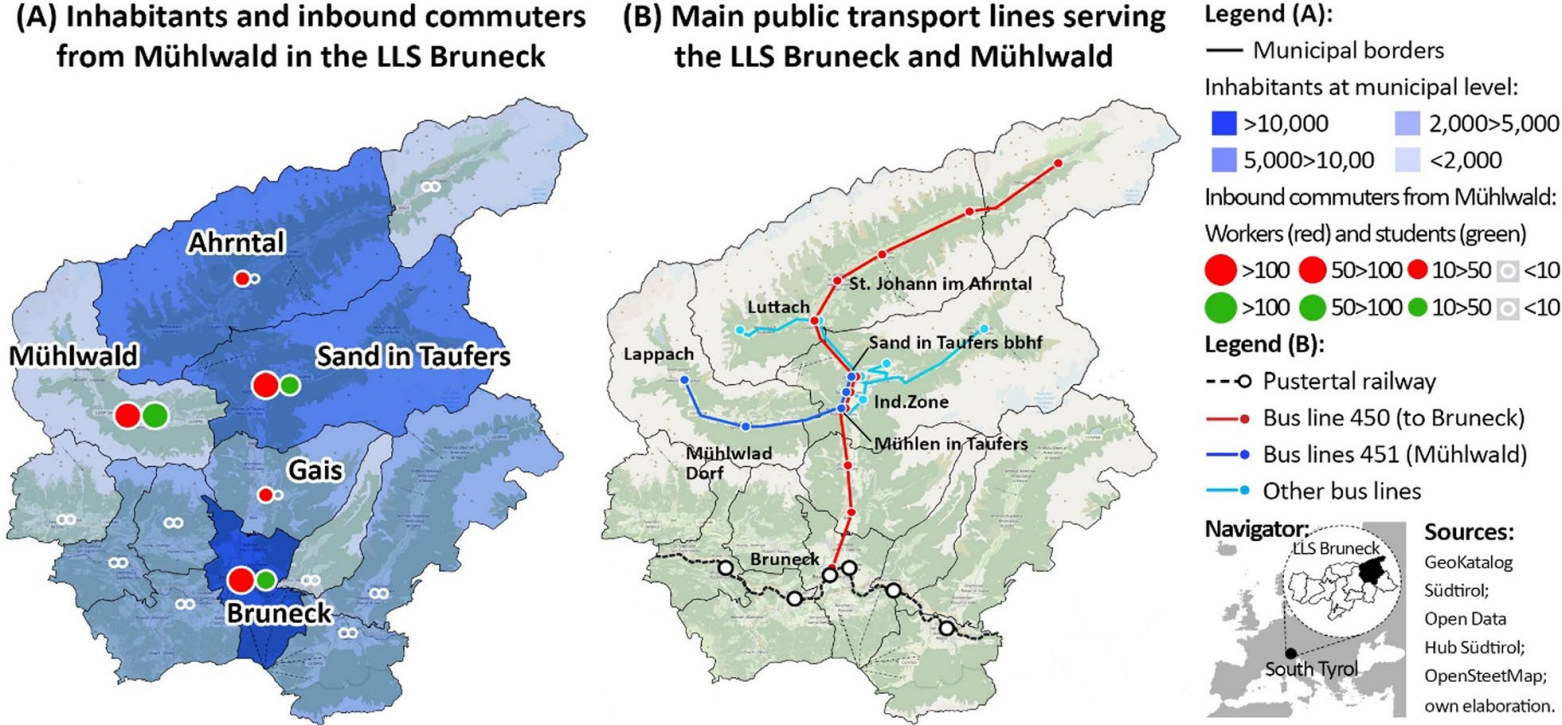
The study area of Mühlwald and the LLS Bruneck

Mühlwald is a remote valley of South Tyrol (Italy) with about 1400 inhabitants and belongs to the local commuting area of Bruneck (LLS Bruneck; [22], Fig. 4). It highly depends on neighbouring municipalities to access workplaces, middle and high schools and general facilities, thus registering a high share of outbound commuters and students [3, 23]. As regards its PT supply, Mühlwald is served by a minor extra-urban bus line crossing the valley once per hour from 6 am to 8 pm [39]. This line connects the valley to a major bus terminus, served by other extra-urban bus lines that lead up to the closest urban hub of Bruneck (ca 20 km away).

In autumn 2022, a telephone travel-diary survey has been carried out with the residents of Mühlwald. A random sampling approach [9] has been followed based on the official list of resident households (ca 500 in total). This has allowed involving 118 individuals randomly. As visible in Table 2, the respondents have been selected to involve a series of accessibility-relevant subgroups. In particular, we defined the sample to least 30 respondents for females, people under the age of 18 and over the age of 65, people belonging to households of at least four members, people working full-time, people living in dispersed hamlets, people mostly travelling by PT, and people having no access to a private car. As visible in Table 2, all these groups have at least 30 respondents, except for people under 18 and over 65, which have been challenging to involve, and people using mostly PT (27 respondents). These sample characteristics allow testing the PT-STA model with a heterogeneous group of rural dwellers supposed to have different accessibility-relevant constraints. Due to the involvement of at least 30 people for the listed subgroups, some restrained sociodemographic differences between the sample and reference population may be noticed (see Table 2. In particular, females are partially overrepresented (57.6% of the sample and 47.7% of the population, as well as people from large households (with an average household size of 3.6 members in the sample, against the population average of 2.7 members; [1, 2]. Conversely, the age and place or residence characteristics are very consistent.

**Table 2:** Characteristics of the sample used to test the PT-STA model and its reference population

Variable types	Variables	Subgroups	Sample in this study (118 members)		Reference population of Mühlwald (1,402 inhabitants)	
			Number	Share	Number	Share
Variables with data available both for the sample and reference population	Gender	Males	50	42.4%	735	52.3%
		Females	68	57.6%	667	47.7%
	Age group	People age < 18	19	16.1%	256	18.3%
		People age 18–65	79	66.9%	903	64.4%
		People age > 65	20	16.9%	243	17.3%
	Place of residence	Built-up rural settlement	72	61.0%	906	64.6%
		Dispersed rural hamlet	46	39.0%	496	35.4%
	Average household size (members)^a^		3.6	–	2.7	–
Variables with data available only for the sample	Household composition	1–2 members	25	21.2%	–	–
		3 members	22	18.6%	–	–
		> 3 members	71	60.2%	–	–
	Employment status	Full-time	41	34.7%	–	–
		Part-time	28	23.7%	–	–
		Not employed or retired	28	23.7%	–	–
		Student	21	17.8%	–	–
	Main mode of transport	Mostly public transport	27	22.9%	–	–
		Mostly private car	58	49.2%	–	–
		Mostly other modes	33	28.0%	–	–
	Private car availability	Always	75	63.6%	–	–
		Sometimes	13	11.0%	–	–
		Never	30	25.4%	–	–

^a^This is the only statistical data regarding the household size available for the population of Mühlwald and the sample

### PT-STA modelling

(1) **STPA**^**PT**^**:** To estimate the STPA^PT^, we rely on the fixed activity couples listed in the travel diaries. Travel time by PT is calculated in ArcGIS by using the GTFS-Dataset of the transport operator STA [33, 39]. This includes walking time to/from stops, waiting time at stops based on schedules, in-vehicle time and time spent in transfers. Values are calculated by assuming individuals to start their trip right after a fixed activity ends and take the first PT connection available with the earliest possible arrival time at the destination. This approach is not always rational, since, in some cases, the first available connection could take a longer travel time than the following one and might not be preferred. However, this issue barely applies to our study areas due to homogenous timetables and travel times. At the same time, this approach allows a good trade-off between computing time and complexity.

(2) **PPA**^**PT**^** and WPPA**^**PT**^**:** To determine the extent of the PPAs^PT^, we define each discretionary location as a 250 × 250 m raster cell. Travel time figures by PT from the first fixed activity to each cell and the following fixed activity are calculated to identify the cells belonging to the PPAs^PT^. As regards the visiting time, we set it to 10 min based on the travel-diary data collected in Mühlwald. This is the average minimum duration of discretionary activities reported by the interviewed people in their diaries. To obtain the WPPAs^PT^, the PT Reachability index (*R*_*j*_) is calculated. Some specifications are needed:

- *Actual performances:* The actual performances of the PT system are estimated in ArcGIS based on the GTFS dataset mentioned above. The total travel time is calculated doo-to-door by considering the actual PT schedules and the sections by walking. The out-of-vehicle travel time focuses on the access and egress time, the waiting time at stops and the transfer time. The travel-chain sections are measured as follows: each PT line separated by the others through a transfer is a single section. In addition, the access and egress time by walking is considered a different section.
- *Ideal performances:* The ideal performances are calculated by taking the private car as a benchmark since this tends to be the quickest transport mean in the study area. The ideal total travel time is calculated as the travel time by car for the observed trip. The ideal out-of-vehicle travel time is assumed to be equal to 1 min for any trip, i.e. a minimum time needed to/from the parking spot to the origin/destination. Finally, the ideal number of travel chain sections is set to 1 for any trip, since the car does not require any transfer or line combination.
- *Weighting factors:* As anticipated in Sect. 2.2.2, overall weighting factors are obtained from a survey conducted with a sample of ca 1800 South Tyrolean residents and ca 400 tourists [34]. Based on the answers given by the residents only, the degree of importance assigned by the sample to several PT attributes has been measured with a 0–100% scale (0% = minimum; 100% = maximum). The examined attributes encompass several aspects, such as the quality of the network, reachability of stops, on-board comfort, service reliability, digital information means, tariffs and ticketing systems. Similarly to previous studies like Cavallaro and Dianin [5], we consider only the attributes in Pechlaner et al. [34] that are directly connected to the PT attributes of our model to get their overall importance rates. For both the *total travel time* (*x*_*1*_ in Table 1) and the *travel-chain sections* (*x*_*3*_ in Table 1), the overall importance rate obtained in Pechlaner et al. [34] is equal to 70% (*W*_*x1,3*_ = 0.70), as reported also in Cavallaro and Dianin [5]. For the *out-of-vehicle travel time*, we combine the rates given to two relevant attributes, namely the time to reach the PT stops and the time for PT transfers. This results in an importance rate of 54% (*W*_*x2*_ = 0.54). The three weighting factors are reported in Annex 1 and applied to the 118 sample members.

(3) **WFOS**^**PT**^**:** Based on PPAs^PT^ and WPPAs^PT^, the discretionary opportunities are counted and weighted to obtain the WFOS^PT^. We rely on the georeferenced database of OpenStreetMap comprising all the amenities in the study area, such as groceries, shopping facilities, healthcare and leisure facilities. We exclude workplaces, schools, and other educational facilities since they typically represent the location of fixed activities. The discretionary opportunities are weighted by means of the public transport Reachability index of the raster cell where they lie.

(4) **STA**^**PT**^_**fix**_**:** The travel-time thresholds needed to obtain STA^PT^_fix_ are inspired by the commuter allowances in force in South Tyrol and Austria [4, 7]. The former focuses on the distance, especially the waiting time to travel to the workplace and return by PT. People travelling more than 18 km per direction with a total waiting time of at least 60 min in their commuting travel chain are entitled to a refund. The Austrian system focuses instead on the entire travel time only, by establishing that people that have a home-to-work commute of more than 60 min in total are eligible for refunding. Based on these examples, we set the travel-time threshold of Mühlwald for “home-to-work” (and vice-versa) travels to 60 min. However, there is no similar reference threshold for “home-to-school” (and vice-versa) and “other errands” travels, and some assumptions are necessary. Considering that educational facilities (especially elementary and middle schools) should be spread across the territory since they are essential facilities for households (e.g. [13, 32]), we set the threshold for “home-to-school travels” to 30 min considering the rural nature of Mühlwald. Conversely, the threshold for “other errand” travels is set to 45 min, since many facilities are often unavailable in small municipalities like Mühlwald (Table 3).

**Table 3:** Travel-time thresholds for the study area of Mühlwald for STA^PT^_fix_ and STA^PT^_dis_

Accessibility indicator	Fixed-activity-couple and discretionary-opportunity types		Reference thresholds	Model-test thresholds	Explanation of the selection of the thresholds
STA^PT^_fix_	*y*_*1*_	Home-to-work	60 min	60 min	Commuter allowances in force in South Tyrol and Austria are taken as a reference for home-to-work travels. The other thresholds are derived accordingly
	*y*_*2*_	Home-to-school	None	30 min	
	*y*_*3*_	Other-errand	None	45 min	
STA^PT^_dis_	*y*_*1*_	Basic retail and pharmacies	15 min	30 min	Car-based thresholds of the PROFECY project are used as a reference and adjusted considering the usually longer travel time by PT
	*y*_*2*_	Healthcare and general facilities	30 min	45 min	
	*y*_*3*_	Leisure and other facilities	45 min	60 min	

(5) **STA**^**PT**^_**dis**_**:** The thresholds for STA^PT^_dis_ are inspired by the EU project PROFECY [14, 35]. To identify inner peripheries in the EU, the project has set the following travel time thresholds for the access to services of general interest by car: 15 min for supermarkets, convenience stores and pharmacies; 30 min for banks, post offices and medical practices; 45 min for leisure facilities like cinemas. Areas not able to meet these thresholds are labelled as inner periphery as regards the transport accessibility to services. In the PROFECY project, Mühlwald is identified as the inner periphery according to its remoteness and distance from main amenities and centres. Therefore, the thresholds set by the PROFECY project may be considered generally suitable for our study area. However, they refer to the travel time by car and thus need a partial adjustment based on the generally longer times by PT. On this basis, the threshold for “basic retail and pharmacies” is set to 30 min; the one for “healthcare and general facilities” is set at 45 min; finally, the threshold for “leisure and other facilities” is of 60 min (Table 3). As further discussed in the limits of this study (Sect. 4.2), identifying these thresholds may be subject to arbitrariness, and it heavily depends on the policy goals placed for the analysis area.

### Results

**Fig. 5:**
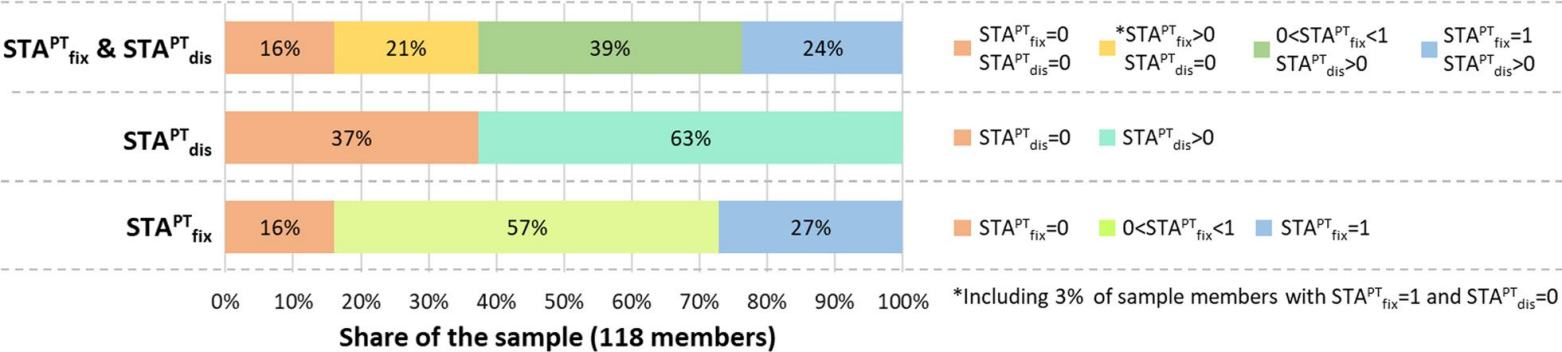
Synthetic results of the PT-STA model in Mühlwald (see Annex 1 for the extended figures)

Figure 5 summarises the results of the PT-STA model at the sample level. Instead, Annex 1 provides an extended and detailed version of the results with one row for each sample member. As visible both in Fig. 5 (first stacked bar) and Annex 1, four main groups of results may be recognised:

- 19 individuals (16% of the sample) register both STA^PT^_fix_ and STA^PT^_dis_ = 0 (highlighted in orange in the first stacked bar of Fig. 5 and in Annex 1), meaning that they can neither perform any daily fixed activities in the given timeframes and thresholds, nor access any discretionary opportunities.
- 25 individuals (21% of the sample) register STA^PT^_fix_ > 0 but their STA^PT^_dis_ = 0 (highlighted in yellow in the first stacked bar of Fig. 5 and in Annex 1). These people can access at least part of their daily fixed activities by PT in compliance with the given timeframes/thresholds. However, they have not enough extra space–time budget to access also discretionary opportunities.
- 46 individuals (39% of the sample) reach both a 0 < STA^PT^_fix_ < 1 and STA^PT^_dis_ > 0 (highlighted in green in the first stacked bar of Fig. 5 and in Annex 1). This means they are able to access part of (but not all) their fixed activities by PT within the timeframes/thresholds, as well as a minimum number of discretionary opportunities in compliance with the given requirements.
- 28 individuals (24% of the sample) have STA^PT^_fix_ = 1 and register also a STA^PT^_dis_ value > 0 (highlighted in blue in the first stacked bar of Fig. 5 and in Annex 1). These are the only people able to reach all their fixed activities within the available timeframes and set thresholds, and visit at least one discretionary opportunity belonging to basic service types within the thresholds.

This last group may be considered as the only one reaching an acceptable minimum degree of PT accessibility both for the access to fixed activities and discretionary opportunities. The discussion of such results is addressed in detail in Sect. 4.1, to point out the added value of the PT-STA model.

**Fig. 6:**
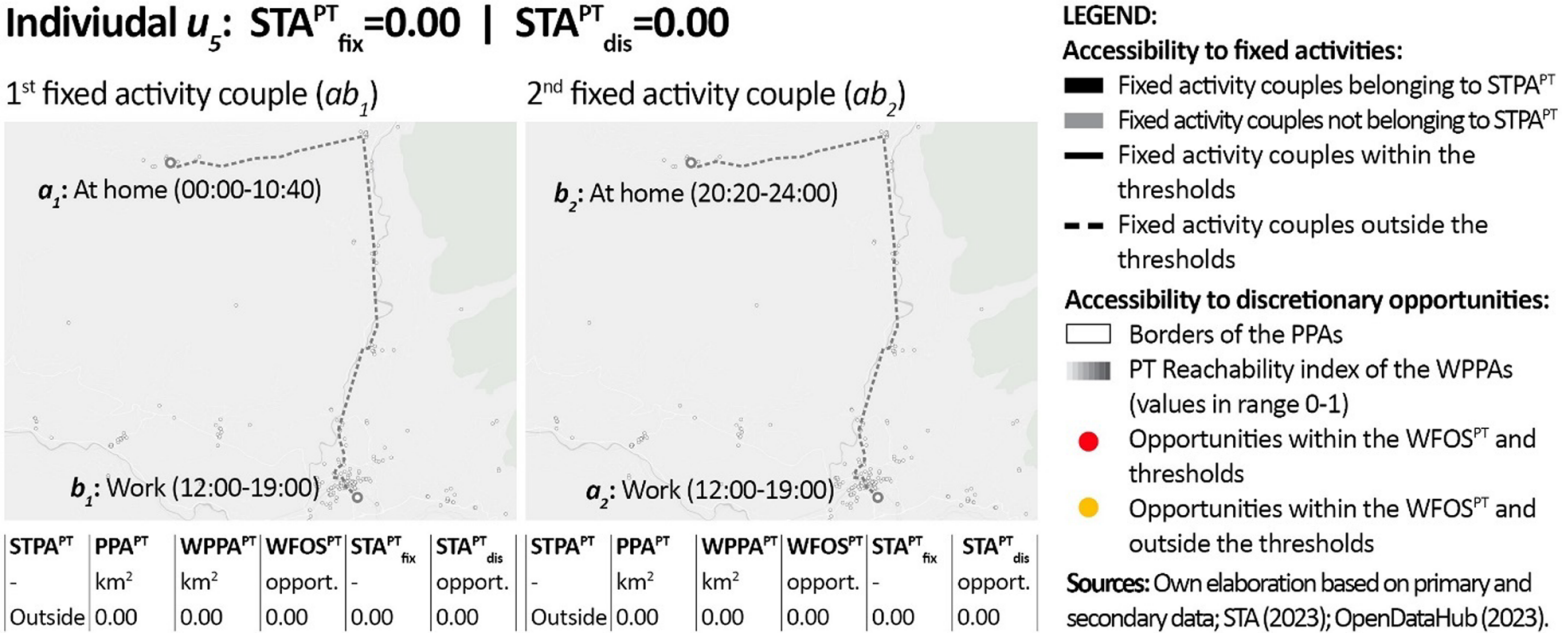
Results of the PT-STA model for *u*_*5*_ (see Annex 1 for details)

**Fig. 7:**
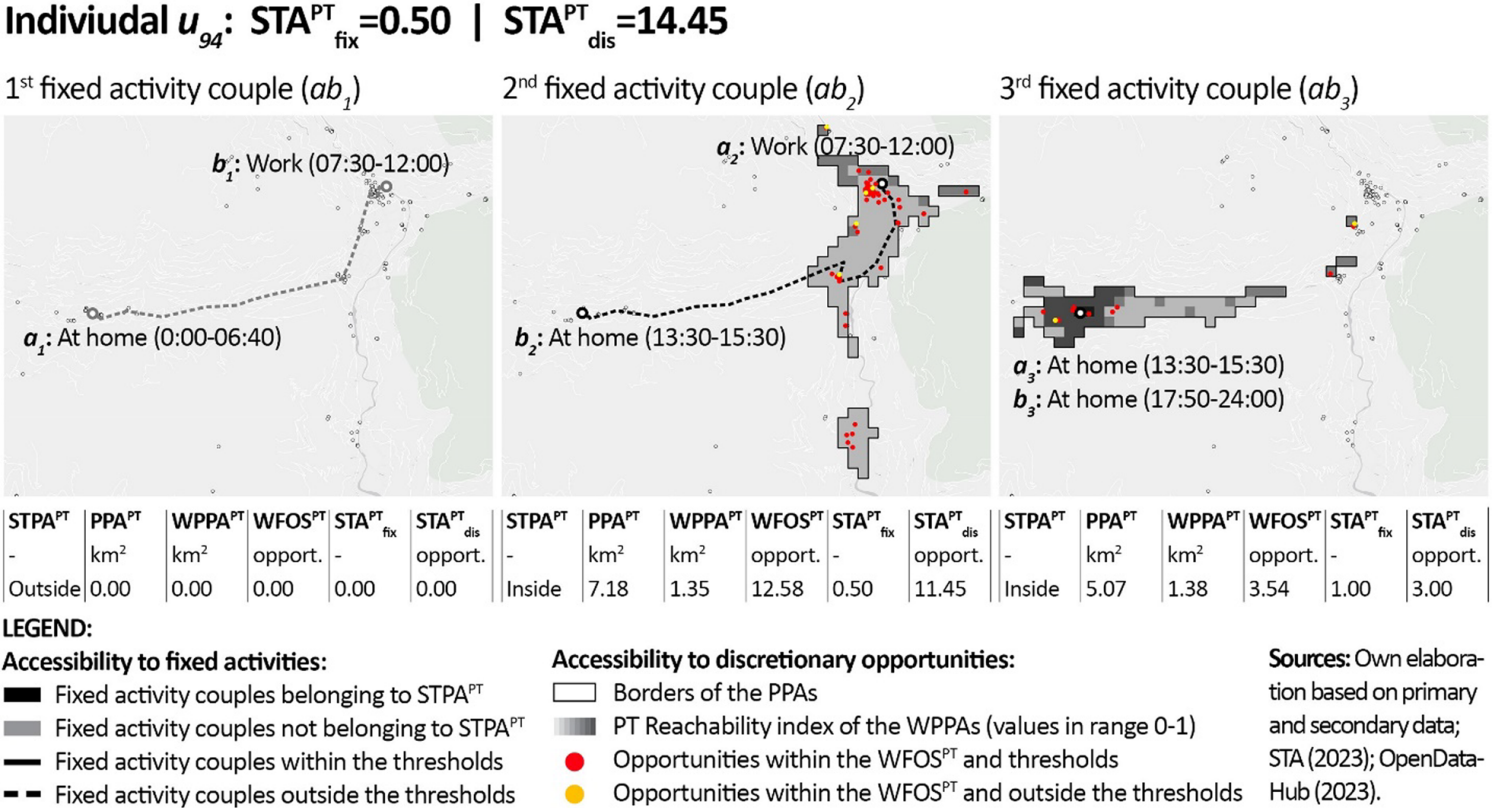
Results of the PT-STA model for *u*_*94*_ (see Annex 1 for details)

**Fig. 8:**
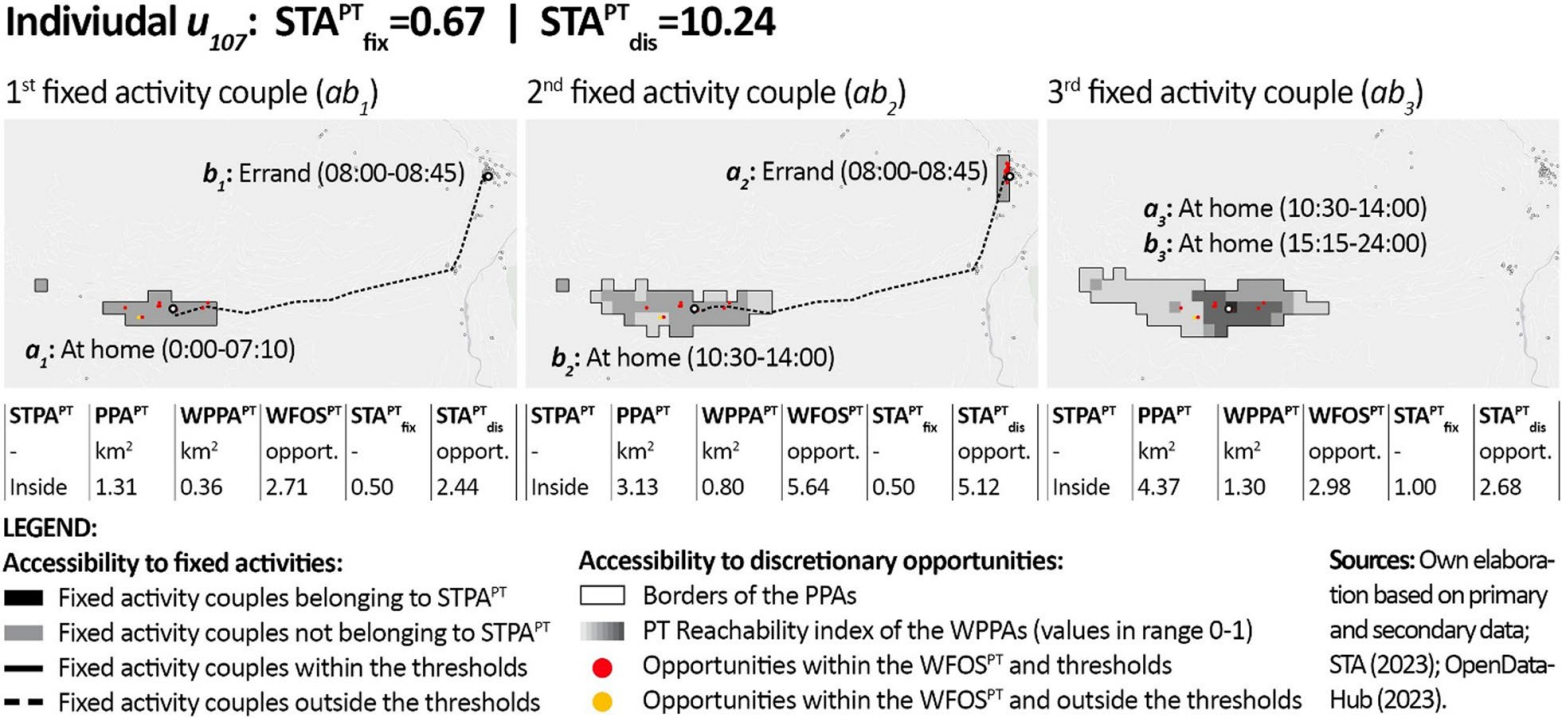
Results of the PT-STA model for *u*_*107*_ (see Annex 1 for details)

In order to provide individual-level details regarding the results, Figs. 6, 7 and 8 show the outputs of the PT-STA model for three sample members in detail, by distinguishing their single fixed activity couples. As visible, these individuals reflect: (1) a case with poor accessibility since no fixed activity couple is feasible by PT; (2) a typical case where part of the fixed activity couples listed in the diary are not feasible by PT; and (3) an ideal case where all the fixed activity couples are feasible by PT. In detail:

Individual *u*_*5*_ (Fig. 6) is a man living in Mühlwald and working full time in Bruneck (see Fig. 4). His daily life includes only two fixed activity couples, namely home-work in the morning (*ab*_*1*_) and vice versa in the evening (*ab*_*2*_). As visible, his working shift is atypical (12:00–19:00) and this negatively impacts his accessibility. Indeed, during the timeframes where he travels, the PT supply is less frequent and thus the overall travel time is longer. Moreover, the final destination he has to reach is not in the nearby of a PT stop, requiring a relatively long walking time. For these reasons, this person is not able to connect any of his fixed activities by PT neither within the available timeframes (both of 80 min), nor within the reference thresholds (both of 60 min). This leads his STA^PT^_fix_ to be equal to 0. As additional consequence, he also has no extra time budget available for visiting discretionary activities in the timeframe between fixed activities, leading also to an STA^PT^_dis_ = 0.

Individual *u*_*94*_ (Fig. 7) is a woman working part-time whose daily routine includes three fixed activity couples. *ab*_*1*_ is neither doable by PT in the available timeframe nor within the reference threshold. *ab*_*2*_ has a broader timeframe at its disposal which is served by a higher PT frequency. As a consequence, this trip is doable by PT and it enables access to discretionary opportunities. Nevertheless, *ab*_*2*_ takes more travel time than the set threshold. Finally, *ab*_*3*_ is a free timespan between two mandatory homestays at lunch and dinner time that allows access to discretionary opportunities. This example makes it evident that the PT-STA model calculates the accessibility generated by each fixed activity couple independently from the others without considering modal interdependencies. Indeed, although *ab*_*1*_ does not belong to STPA^PT^, the accessibility of *ab*_*2*_ is computed and not assumed equal to 0. Although this approach is not strictly related to the expectable modal choices, it is coherent with the idea of accessibility as potential [20], i.e. a supply-oriented measure of what could be accessible by PT for each single activity couple.

Individual *u*_*107*_ (Fig. 8) is a pensioner who performs three activities daily considered as fixed: a personal errand in the early morning, one mandatory homestay around lunchtime, and a second fixed homestay from the afternoon till the end of the day. Due to his limited space–time constraints, all his fixed activity couples (*ab*_*1–3*_) are feasible by PT in the available timeframe and produce access to discretionary opportunities. However, since he often travels during off-peak hours, the time requested by PT for the fixed trips of *ab*_*1*_ and *ab*_*2*_ is longer than the set thresholds, with a negative influence on his access to fixed activities.

## Discussion

### Insights and added value of the PT-STA model

**Fig. 9:**
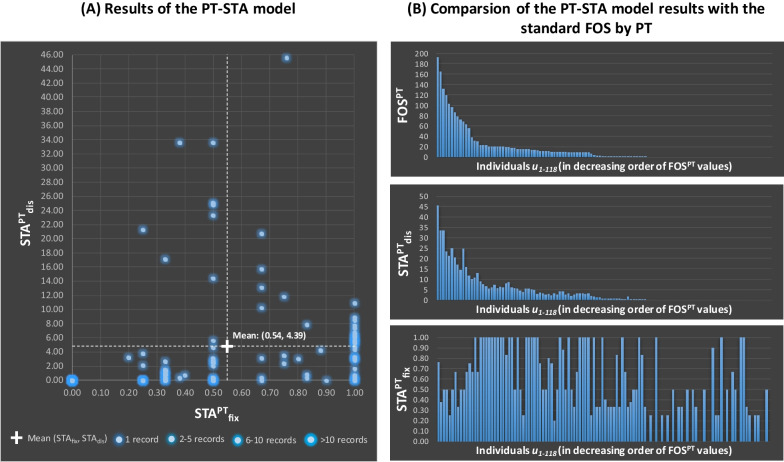
Summary of the PT-STA model results and comparison with the standard FOS^PT^

Figure 9A shows the results of STA^PT^_fix_ and STA^PT^_dis_ in a bivariate graph where each data point is a single sample member. As visible in the graph, there is no evident (linear) correlation between the STA^PT^_fix_ and STA^PT^_dis_ trends over the sample. For instance, ca 21% of the sample members register a STA^PT^_fix_ over the average (0.54) and a STA^PT^_dis_ under the average (4.39) as visible in the bottom-right area of the graph. Conversely, 8% show the opposite trend, as represented in the top-left area of the graph. Such absence of an evident relation between the results of the two accessibility indicators for the sample members is confirmed by the weak correlation value reported in Table 4: Pearson Correlation Coefficient, PCC =  + 0.20. Moreover, the results of STA^PT^_dis_ are much more dispersed than STA^PT^_fix_ (see Table 4, Coefficient of Variation, CV = 176% and 66% respectively).

**Table 4:** Mean, dispersion and correlation of STA^PT^_fix_, STA^PT^_dis_ and of the standard FOS^PT^

	Mean	Standard deviation (STDEV)	Coefficient of variation (CV) (%)	Pearson correlation coefficient (PCC)		
				STA^PT^_fix_	STA^PT^_dis_	FOS^PT^
STA^PT^_fix_	0.54	0.36	66	+ 1.00	+ 0.20	+ 0.13
STA^PT^_dis_	4.39	7.74	176	–	+ 1.00	+ 0.98
FOS^PT^	16.45	32.95	200	–	–	+ 1.00

The differences between STA^PT^_fix_ and STA^PT^_dis_ are particularly visible for members of the sample like pensioners and homemakers, who tend to have a few mandatory activities typically performed either at home or in their surroundings (leading to high STA^PT^_fix_). However, this tendency to stay in the vicinity of their home leads to limited access to discretionary opportunities in a remote place such as Mühlwald (i.e. low STA^PT^_dis_ values). The opposite applies to sample members with tight daily schedules but daily travelling to urban hubs with many amenities (like some adult commuters). In their case, the accessibility to fixed activity (STA^PT^_fix_) tends to be low, while their access to discretionary opportunities (STA^PT^_dis_) is relatively high.

The STA^PT^_fix_ and STA^PT^_dis_ differ also from the standard FOS (calculated by considering PT only and called FOS^PT^). This is visible in Fig. 9B, which reports the FOS^PT^, STA^PT^_dis_ and STA^PT^_fix_ results of all the 118 sample members in three column graphs, with the sample members always sorted in decreasing order of FOS^PT^ values. As expectable and visually evident in the graphs, STA^PT^_dis_ and FOS^PT^ are strongly correlated (PCC =  + 0.98 in Table 4), although the magnitude of results is sensibly different due to the introduction of weights and thresholds. Conversely, STA^PT^_fix_ and FOS^PT^ are even less correlated than with STA^PT^_dis_ (PCC =  + 0.13 in Table 4), confirming that the accessibility to fixed activities describes a complementary and not overlapped side of accessibility.

These results highlight the main added values of the PT-STA model. First, the model combines two components (STA^PT^_fix_ and STA^PT^_dis_) that tell complementary stories regarding the PT accessibility. Both of them have to be considered to get a more complete picture of the most relevant PT accessibility issues experienced by different members of the population and address them with more targeted policies. Second, the model integrates thresholds in STA^PT^_fix_ taking into account not only the timeframes actually at the disposal of people but also reference travel-time standards that the PT system should guarantee. This provides a more complete assessment of the degree of access to fixed activities. Third, the model counts in STA^PT^_dis_ only the basic amenities reachable within reference thresholds and weights their relevance based on their degree of PT reachability. This allows focusing only on the basic discretionary opportunities to which the PT system guarantees a minimum degree of access.

The outputs of the PT-STA model may be used by policymakers in different ways. In particular, two evaluations of the presented results may be particularly useful for PT planning:

- Share of people with STA^PT^_fix_ ≠ 1. These individuals cannot perform all their fixed daily trips by PT within the available timeframes and set thresholds. This condition may heavily prevent them from using PT not only for those fixed activities unfeasible by PT, but also for all the other fixed activities directly chained to them. Moreover, it would affect their possibility to visit discretionary opportunities by PT along the way between two fixed activities, by making the potential generated by STA^PT^_dis_ not exploitable by PT. In the case of Mühlwald, this condition affects 73% of the respondents. Policymakers may focus on this share to verify how different PT interventions may reduce it and to what degree, and thus enable more people to use PT for their daily needs.
- Share of people with STA^PT^_dis_ = 0. These people have no access by PT to any basic amenity within their available timeframes and the set thresholds. This is a relevant shortcoming since access to fundamental amenities like basic retail and healthcare are considered essential factors for people's well-being. In Mühlwald, ca 37% of the respondents experience this condition and policymakers may verify which PT interventions are most promising in reducing such share and increasing the access to basic services.

By controlling these and other aspects of STA^PT^_fix_ and STA^PT^_dis_, policymakers may better assess the pros and cons of alternative PT measures, and select the most desirable intervention given both components.

### Limits of the PT-STA model

The main limits of the PT-STA model and its test in this study regard the following aspects: (1) accessibility as potential, (2) travel-time thresholds, (3) timetable flexibility, (4) sample size, (5) generalisation, (6) service frequency, and (7) weighting factors.

(1) *Accessibility as potential*: The fact that the opportunities included in the FOS merely represent a potential in which there might not be any interest by the individual is a general criticism of the concept of the STA. The results of our model, especially in the area of ​​discretionary opportunities, are based on the summary of the potential of all subsequent fixed activities of a person, which can be covered in the specified timeframe. This implies that our analysis has not considered that the impossibility to use PT for at least one of the fixed daily activities (a condition that affects 73% of our sample) could affect all the other modal choices, so preventing the usage of PT for other daily trips and decreasing the PT accessibility results. As mentioned in Sect. 2.2.1, we have taken this approach since accessibility is traditionally defined as a measure of “the potential of opportunities for interaction” [20, 21]. Accordingly, the PT-STA model aims to capture the PT potential beyond the actual modal choices. To meet this target, each fixed activity couple can generate an own PT accessibility potential. This allows pointing out accessibility differences between individuals unable to perform any daily activities by PT, and individuals experiencing such obstacles only for one or some of their daily activities. In this light, our results should be interpreted only in terms of potential accessibility by PT.

(2) *Travel-time thresholds*: Even if the travel-time thresholds selected for this study are derived from political specifications and EU-project findings, they are still based on normative specifications. They are subject to valuations, which may result in different travel time thresholds under different framework conditions in different places. A case-by-case justification of the thresholds is needed for the validation of the procedure and should not always be accepted without reflection.

(3) *Timetable flexibility*: In various cases, the timeframes between subsequent fixed activities are mentioned by respondents with the usually used traffic mode in mind (rather than by focusing only on the mandatory duration of the activities). In cases where the car is usually chosen, this may result in the underestimation of the timeframe length, with negative impacts on PT accessibility. To take this fact into account, the model has considered a flexibility of the timeframes, especially for activities that are usually subject to higher flexibility like the first and last homestays at the beginning and end of the day. Nevertheless, the problem of potentially too short timeframes could have affected the results, resulting in a partial underestimation of the PT accessibility especially for fixed activities.

(4) *Sample size*: Our limited sample size is firstly linked to the small population of Mühlwald. Indeed, our 118 sample members represent almost 10% of the reference population. A second issue is the length and complexity of the travel-diary survey, which discourages a larger participation. On average, about 20 min have been necessary to introduce the survey, reconstruct the diary, ask for sociodemographic information, as well as for individual PT preferences. This length makes it difficult to involve a higher share of the population, which is a common challenge also in other STA studies with relatively small samples if compared with their reference population [6, 40]. Moreover, countries making similar kinds of survey at the governmental level (like Austria) tend to collect a sample from all over the country. This makes the data unsuitable to perform local analyses oriented to PT development as that one presented in the study. Therefore, further work is needed to simplify the travel diary survey as much as possible, and increase the interest of regional and local authorities in the collection of such kind of data for future usages.

(5) *Generalisation*: The limited sample size implies that no statistically generalizable statement is possible. However, the purpose of this study is to present and test the PT-STA model so as to compare it with the standard STA and point out its potential. For this reason, we have decided to test the model in a second rural area for which travel-diary data has been collected: the municipality of Sooß in Lower Austria. Even in this case, we deal with a rural area with a small population and sample (ca 1100 inhabitants and 104 respondents). As visible in Table 5, the results of Sooß are consistent with those of Mühlwald presented in Table 4. Specifically, STA^PT^_fix_ and STA^PT^_dis_ have a weak correlation also in Sooß (+ 0.11 in Sooß and + 0.20 in Mühlwald), and this correlation is even weaker between STA^PT^_fix_ and FOS^PT^ (+ 0.05 in Sooß and + 0.13 in Mühlwald). Instead, the STA^PT^_dis_ and FOS^PT^ results are coherent but different in magnitude in both areas (correlation of + 0.99 in Sooß and + 0.98 in Mühlwald). Although this second test does not enable a statistical generalisation of the single case studies, it shows how the added value of the PT-STA visible with the test in Mühlwald is also visible in the second test of Sooß. From the perspective of policy makers, this is a positive signal of the potential usefulness of the PT-STA model, although larger samples are needed in future case-study applications to reach statistically reliable results.

**Table 5:** Results of the second test of the PT-STA model in Sooß (Lower Austria)

	Mean	Standard deviation (STDEV)	Coefficient of variation (CV) (%)	Pearson correlation coefficient (PCC)		
				STA^PT^_fix_	STA^PT^_dis_	FOS^PT^
STA^PT^_fix_	0.24	0.35	150	+ 1.00	+ 0.11	+ 0.05
STA^PT^_dis_	17.21	47.93	278	–	+ 1.00	+ 0.99
FOS^PT^	76.45	246.29	322	–	–	+ 1.00

(6) *Service frequency*: This study focuses on the earliest and fastest possible connection in the given timeframe of each pair of subsequent fixed activities. To enhance the model, all possible connections of every couple of subsequent fixed activities could be examined. In this way, a qualitative distinction between different fixed-activity connections can be made, not only in terms of whether a destination is reached or not, but also how often the destination can be reached in a timeframe. This would allow integration a certain degree of flexibility in travel planning, which can represent a relevant factor for the individual assessment of a connection, especially when it comes to comparing it to private cars. The comparison can be used for a weighting of the results, whereby the integration of a flexibility criterion to the analysed PT attributes in the form of frequency determination would further improve the model.

(7) *Weighting factors*: The weighting factors used in our case study have been applied to the whole sample based on a large survey involving ca 1800 South Tyrolean residents. A customisation of the weighting factors either by sociodemographic groups or by single respondents could potentially increase the person-based quality of the model. However, such kind of customisation is not viable in our study areas since we have neither sociodemographic variants of the overall importance rates by Pechlaner et al. [34], nor individual interval-scale rates from our sample members in Mühlwald. On the one side, this may be considered a weakness for our person-based approach. On the other side, the usage of overall weights derived from a large sample contributes to the generalization of the accessibility results, which is relevant for the usefulness of our model for policymaking.

## Conclusions

This paper has proposed an integration of the standard STA measure (called PT-STA model) to meet three targets: (1) focusing on PT accessibility, (2) measuring accessibility to both fixed and discretionary activities; and (3) integrating travel-time thresholds. These three targets have been addressed by adjusting the way of defining the STPA, introducing an index to weight both the PPAs and FOS, and by integrating travel-time thresholds in the two outputs of the model: the STA^PT^_fix_ and STA^PT^_dis_ indicators. The PT-STA model has been tested with a sample of 118 people living in the rural valley of Mühlwald, in South Tyrol. The results of the test suggest that the PT-STA model provides two complementary and not overlapped viewpoints on the accessibility to fixed activities and discretionary opportunities. Policymakers may benefit from the integration of both in the assessment of PT interventions. Nevertheless, some conceptual and operational caveats must also be considered, as described in Sect. 4.2 above.

Despite such limitations, the PT-STA model may represent a useful tool for researchers and practitioners to assess the impacts of PT interventions on the accessibility of single individuals or specific groups to both fixed activities and discretionary opportunities. First, the PT-STA model requires defining travel-time thresholds based on policy goals and reference standards that are desirable for the area of analysis. This may be useful for policymakers, who must reflect on minimum standard conditions that the PT system is asked to guarantee. Second, the PT-STA model may allow an understanding of to what extent a PT intervention may be beneficial for access to fixed activities, discretionary opportunities or both. This allows observing the impacts of PT interventions from two complementary points of view and selecting the most suitable intervention depending on the specific policy goal or the best trade-off between the two accessibility dimensions. Third, the PT-STA model allows integrating access to both fixed and discretionary activities in transport equality evaluations. Indeed, the effect of a specific PT intervention on a population subgroup (such as the elderly, women or members of large households) may be compared to the impact on the overall population. This is also possible with traditional STA measures. Still, the PT-STA model allows doing it with a focus on PT only and broadening the evaluation to both the fixed and discretionary dimensions of accessibility. Finally, the analysis of specific indicators as the share of people with STA^PT^_fix_ ≠ 1 and STA^PT^_dis_ = 0 suggested in Sect. 4.1 may enable further equity analyses of the accessibility outputs that go beyond distributional analyses, such as minimum-standard assessments (see e.g. [28].

Considering these potentials, the PT-STA model may be considered a valuable contribution to the space–time accessibility debate, and it may be a useful tool to foster person—(and not only place-) based evaluations of the accessibility implications of PT measures.

## Figures and Tables

**Table:**
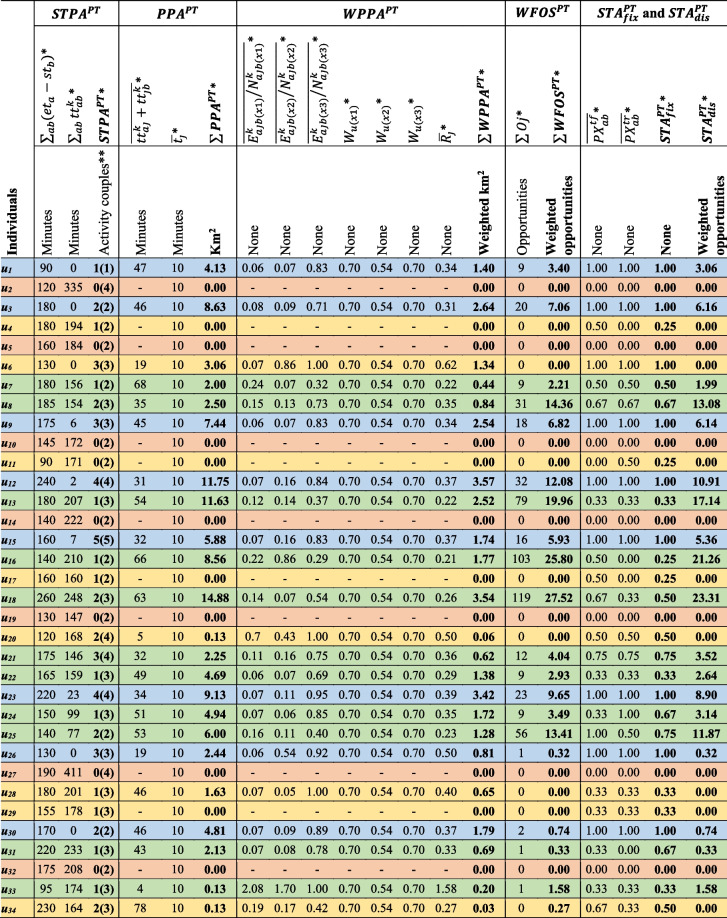

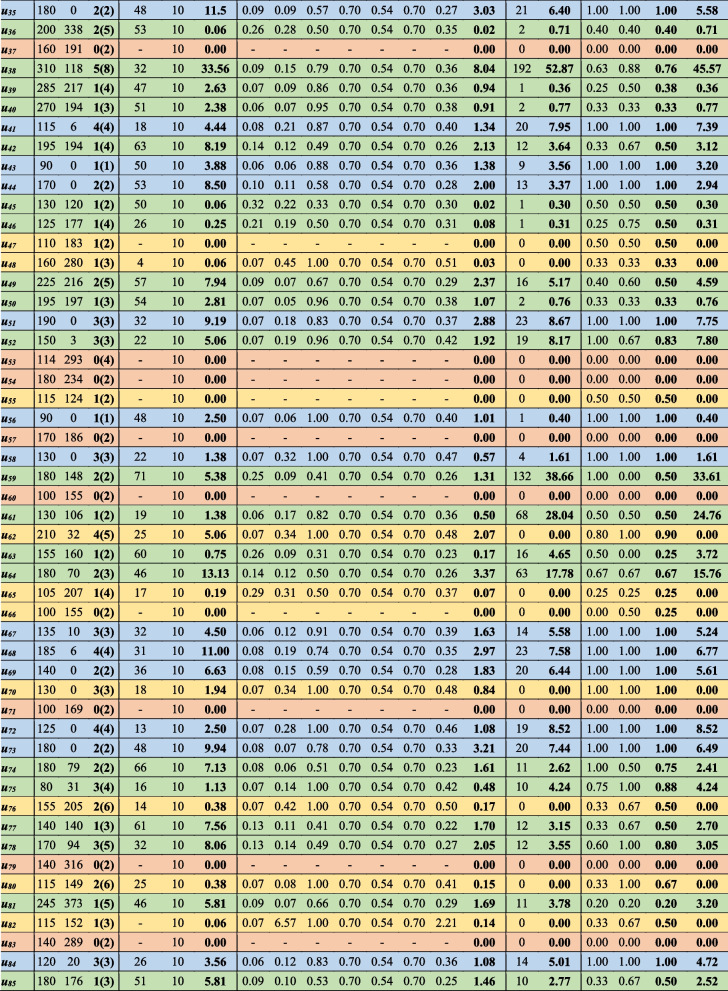

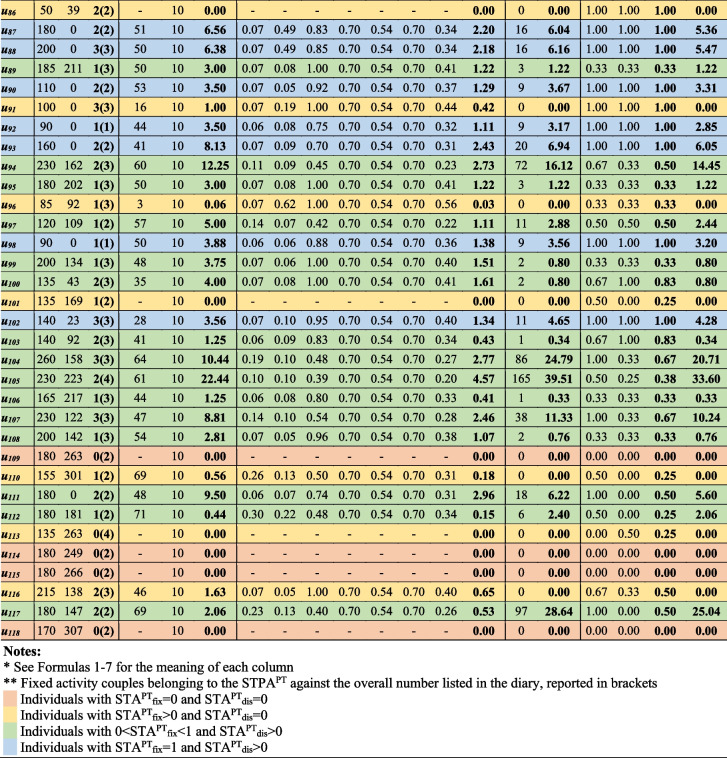
Annex 1: Results of the PT-STA model for all the sample members.

## Data Availability

The dataset supporting the conclusions of this article is available in the ReposiTUm repository. The full (anonymised) dataset of the travel-diary survey is available at the following persistent identifier: 10.48436/hq7b7-xsa12. The extended version of the STA-fix and STA-dis results of Mühlwald with the related intermediate steps are available at the following persistent identifier: 10.48436/k1ce7-hrt53. The same applies to the case study of Sooß (same persistent identifier).

## Footnotes

1 Work, Homestay, Business, Education, Escorting people, Shopping, Care of relatives, Other private errands, Leisure, Other.

2 For instance, a sport training that steadily occurs at a given place and time on daily basis may be classified as “leisure” (activity category) and labelled as “fixed”, since it is considered and treated as such in the daily life of an individual.

3 GTFS (General Transit Feed Specification) is a common format for PT schedules and related geographic information.

## References

[1] Astat. (2023). Daticomunali.qvw [WWW Document]. Retrieved 22 May, 2023, from https://qlikview.services.siag.it/QvAJAXZfc/opendoc_notool.htm?document=Daticomunali.qvw&host=QVS%40titan-a&anonymous=true.

[2] Astat. (2023). Statistisches Gemeindeprofil - 088 Mühlwald. Bolzano, Italy.

[3] Astat. (2022). Mobilität und Verkehr in Südtirol 2019 (No. 234). PROVINCIA AUTONOMA DI BOLZANO - ALTO ADIGE Istituto provinciale di statistica Rip. 12 - Servizio Strade Rip. 38 - Mobilit, Bolzano, Italy.

[4] Bundesministerium Finanzen AT. (2022). Unzumutbarkeit der Benützung von Massenverkehrsmitteln [WWW Document]. Retrieved 14 December, 2022, from https://bmf.gv.at/themen/steuern/arbeitnehmerinnenveranlagung/pendlerfoerderung-das-pendlerpauschale/unzumutbarkeit-benuetzung-massenverkehrsmittel.html.

[5] Cavallaro F, Dianin A (2020). An innovative model to estimate the accessibility of a destination by public transport. Transportation Research Part: Transport and Environment.

[6] Chen Z, Yeh AG-O (2021). Socioeconomic variations and disparity in space–time accessibility in suburban China: A case study of Guangzhou. Urban Studies.

[7] CIVIS.BZ. (2023). Servizio | CIVIS, la nuova Rete Civica dell’Alto Adige: Pendolari – contributi per spese di viaggio a favore di lavoratrici e lavoratori dipendenti [WWW Document]. Retrieved 26 April, 2023, from https://civis.bz.it/it/servizi/servizio.html?id=1010540.

[8] Curtis C, Ellder E, Scheurer J (2018). Public transport accessibility tools matter: A case study of Gothenburg, Sweden. Case Studies on Transport Policy.

[9] Daniel J (2012). Sampling essentials: Practical guidelines for making sampling choices.

[10] DfT. (2013). Accessibility indicators [WWW Document]. Retrieved 13 March, 2023, from https://www.data.gov.uk/dataset/6ce25e42-bdac-4a6c-9d75-736c7e7d1139/accessibility-indicators.

[11] Dianin A, Gidam M, Hauger G (2022). Isolating the role of the transport system in individual accessibility differences: A space-time transport performance measure. Applied Sciences.

[12] EC. (2021). *Sustainable & smart mobility strategy: Putting European transport on track for the future*. European Commission.

[13] EC. (2011). *Study on Social Services of General Interest*. European Commission - DG for Employment, Social Affairs and Inclusion.

[14] ESPON. (2017). *PROFECY—inner peripheries: National territories facing challenges of access to basic services of general interest* [WWW Document]. ESPON. Retrieved 11 January, 2023, from https://www.espon.eu/inner-peripheries.

[15] Fransen K, Farber S, Lucas K, Martens K, Di Ciommo F, Dupont-Kieffer A (2019). 4-Using person-based accessibility measures to assess the equity of transport systems. Measuring transport equity.

[16] Geurs KT, van Wee B (2004). Accessibility evaluation of land-use and transport strategies: Review and research directions. Journal of Transport Geography.

[17] Gutiérrez J, Monzón A, Piñero JM (1998). Accessibility, network efficiency, and transport infrastructure planning. Environment and Planning A: Economy and Space.

[18] Hägerstrand T (1970). What about people in Regional Science?. Papers of the Regional Science Association.

[19] Handy SL, Niemeier DA (1997). Measuring accessibility: An exploration of issues and alternatives. Environment and Planning A: Economy and Space.

[20] Hansen WG (1959). How accessibility shapes land use. Journal of the American Institute of Planners.

[21] Ingram DR (1971). The concept of accessibility: A search for an operational form. Regional Studies.

[22] ISTAT. (2015). Sistemi Locali del Lavoro: Nota metodologica, Statistiche report. Istat.

[23] ISTAT. 2011. Istat.it - Sistemi locali del lavoro [WWW Document]. Retrieved 18 March, 2021, from https://www.istat.it/it/informazioni-territoriali-e-cartografiche/sistemi-locali-del-lavoro.

[24] Kwan M-P (1999). Gender and individual access to urban opportunities: A study using space–time measures. The Professional Geographer.

[25] Kwan M-P (1998). Space-time and integral measures of individual accessibility: A comparative analysis using a point-based framework. Geographical Analysis.

[26] Kwan M-P, Weber J (2003). Individual accessibility revisited: Implications for geographical analysis in the twenty-first century. Geographical Analysis.

[27] Lee J, Miller HJ (2018). Measuring the impacts of new public transit services on space-time accessibility: An analysis of transit system redesign and new bus rapid transit in Columbus, Ohio, USA. Applied Geography.

[28] Martens K, Bastiaanssen J, Lucas K, Lucas K, Martens K, Di Ciommo F, Dupont-Kieffer A (2019). 2-Measuring transport equity: Key components, framings and metrics. Measuring transport equity.

[29] Miller EJ (2018). Accessibility: Measurement and application in transportation planning. Transport Reviews.

[30] Miller HJ, Levinson DM, Krizek KJ (2005). Place-based versus people-based accessibility. Access to destinations.

[31] Miller HJ (1999). Measuring space-time accessibility benefits within transportation networks: basic theory and computational procedures. Geographical Analysis.

[32] Moreno-Monroy AI, Lovelace R, Ramos FR (2018). Public transport and school location impacts on educational inequalities: Insights from São Paulo. Journal of Transport Geography.

[33] Open Data Hub. (2023). Open Data Hub - Develop digital solutions based on real data [WWW Document]. Open Data Hub - Dev. Digit. Solut. Based Real Data. Retrieved 26 April, 2023, from https://opendatahub.com/.

[34] Pechlaner, H., Scuttari, A., Martini, M., & Bonelli, A. (2015). Analisi della soddisfazione del trasporto su gomma. Eurac Research - Istituto per lo Sviluppo Regionale e il Management del Territorio, Bolzano, Italy.

[35] Schürmann, C. (2017). PROFECY—Processes, Features and Cycles of Inner Peripheries in Europe: Annex 7. Delineation 3—Series of Maps illustrating the Delineation Process. ESPON EGTC.

[36] Schwanen T, Kwan MP, Ren F (2008). How fixed is fixed? Gendered rigidity of space-time constraints and geographies of everyday activities. Geoforum.

[37] Sinha KC, Labi S (2007). Transportation decision making.

[38] Smith N, Hirsch D, Davis A (2012). Accessibility and capability: The minimum transport needs and costs of rural households. Journal of Transport Geography.

[39] STA. (2022). Fahrplantabellen [WWW Document]. Retrieved 13 July, 2022, from www.suedtirolmobil.info. https://www.suedtirolmobil.info/de/meine-fahrt/fahrplantabellen.

[40] Stopher PR (1992). Use of an activity-based diary to collect household travel data. Transportation.

[41] Stopher, P. R., & Metcalf, H. M. A. (1996). Methods for household travel surveys. NCHRP Synth. Highw. Pract.

[42] UN. (2021). THE 17 GOALS|Sustainable Development [WWW Document]. Retrieved 10 December, 2021, from https://sdgs.un.org/goals.

[43] van Wee, B., & Mouter, N. (2020). Evaluating transport equity. In *Advances in transport policy and planning*. Academic Press.

[44] Young W, Tilley F (2006). Can businesses move beyond efficiency? The shift toward effectiveness and equity in the corporate sustainability debate. Business Strategy and the Environment.

